# Draft Genome Sequence of Nocardia seriolae Strain N-2927 (NBRC 110360), Isolated as the Causal Agent of Nocardiosis of Yellowtail (Seriola quinqueradiata) in Kochi Prefecture, Japan

**DOI:** 10.1128/genomeA.00082-15

**Published:** 2015-03-12

**Authors:** Masayuki Imajoh, Yoichi Fukumoto, Jin Yamane, Masaki Sukeda, Masato Shimizu, Kouhei Ohnishi, Syun-ichirou Oshima

**Affiliations:** a Department of Aquaculture, Fish Disease Laboratory, Kochi University, Nankoku, Kochi, Japan; b Graduate School of Kuroshio Science, Kochi University, Nankoku, Kochi, Japan; c Research Institute of Molecular Genetics, Kochi University, Nankoku, Kochi, Japan

## Abstract

We report the draft genome sequence of Nocardia seriolae strain N-2927 (NBRC 110360), isolated from cultured yellowtail Seriola quinqueradiata. RAST annotation of the genome revealed 117 genes involved in the virulence, disease, and defense subsystem. Eleven of these genes were predicted as antibiotic resistance genes.

## GENOME ANNOUNCEMENT

Nocardia spp. are aerobic, nonmotile, Gram-positive, bacillary, branching bacteria that belong to the *Actinomycete*s, and more than 50 species have been identified to date (1). Some of these species cause nocardiosis in aquatic organisms (2). The occurrence of nocardiosis in marine fish was first reported in 1967 in yellowtail Seriola quinqueradiata and amberjack Seriola dumerili cultured in Owase, which is located in the southeastern part of Kii Peninsula in Japan (3). The causative agent was named Nocardia seriolae (4). Nocardiosis caused by N. seriolae is a major disease in cultured yellowtail, which accounts for nearly 60% of total marine aquaculture production in Japan (5). Here, we report the draft genome sequence of N. seriolae strain N-2927, isolated from the spleen of diseased yellowtail collected from an aquaculture farm in Kochi Prefecture, Japan, in 2007. This isolate has been deposited in the NITE Biological Resource Center (NBRC, Japan) under code NBRC 110360.

The genomic DNA of N-2927 was extracted and purified with a Qiagen Genomic-tip 500/G kit and a genomic DNA buffer set (Qiagen) according to the method described previously (6). Genome sequencing was performed on a 454-GS Junior System (Roche). A total of 167,713 reads were generated. The obtained reads were combined with the Illumina reads from the NCBI Sequence Read Archive (accession no. DRX020602), and assembled with GS *de novo* assembler version 2.9 software (Roche). The assembly consists of 339 large contigs (>500 bp) with an *N*_50_ value of 45,841 bp and a largest contig size of 127,534 bp. The draft genome sequence of N-2927 has a total of 7,758,286 bp with a G+C content of 68.3%. The draft genome sequence was annotated using the Microbial Genome Annotation Pipeline (http://www.migap.org), yielding 7,531 protein-coding sequences (CDS), 63 tRNA genes, and 3 rRNA operons. The Rapid Annotations using Subsystems Technology (RAST) server (7) was also used for subsystem descriptions. The annotation identified 117 genes involved in the virulence, disease, and defense subsystem. Eleven of these genes were predicted as antibiotic resistance genes. This is interesting, as N-2927 shows multidrug resistance (unpublished data). In addition, the RAST server identified the following subsystems: 594 in carbohydrates; 576 in amino acids and derivatives; 404 in cofactors, vitamins, prosthetic groups, or pigments; 350 in fatty acids, lipids, and isoprenoids; 314 in protein metabolism; 169 in respiration; 145 in DNA metabolism; 126 in nucleosides and nucleotides; 119 in stress response; 107 in RNA metabolism; 65 in regulation and cell signaling; 61 in cell wall and capsule; 55 in sulfur metabolism; 54 in membrane transport; 47 in phosphorus metabolism; 45 in miscellaneous; 40 in nitrogen metabolism; 40 in metabolism of aromatic compounds; 21 in potassium metabolism; 19 in cell division and cell cycle; 15 in iron acquisition and metabolism; 11 in phages, prophages, transposable elements, or plasmids; 4 in secondary metabolism; 3 in dormancy and sporulation; and 2 in motility and chemotaxis.

### Nucleotide sequence accession numbers.

The draft genome sequence of N-2927 has been deposited at DDBJ/EMBL/GenBank under the accession number BAWD00000000. The version described in this paper is the second version, BAWD02000000.
